# Clinical Characteristics and Risk Factors of Lung Cancer-Associated Acute Ischemic Stroke

**DOI:** 10.1155/2019/6021037

**Published:** 2019-12-17

**Authors:** Jiacai Lin, Siting Wu, Rui Xu, Qiang Shi, Chenglin Tian, Fang Cui, Xue Shao, Hui Liu

**Affiliations:** ^1^Department of Neurology, Hainan Hospital of Chinese PLA General Hospital, Sanya 572013, China; ^2^Department of Oncology, Hainan Hospital of Chinese PLA General Hospital, Sanya 572013, China

## Abstract

**Objective:**

To research the clinical characteristics and risk factors of lung cancer-associated acute ischemic stroke (LCA-AIS).

**Methods:**

Patients diagnosed with LCA-AIS, simple lung cancer, and simple AIS were enrolled. The primary information, laboratory results, tumor histopathology, neurological deficits, and survival time of the patients were collected and analyzed.

**Results:**

(1) In the LCA-AIS group, the pathology of 69.56% patients were adenocarcinoma, and the proportion of poorly differentiated patients was significantly more than that in moderately differentiated or highly differentiated. The number of stage IV lung cancer patients in the LCA-AIS group was significantly more common than in other stages. (2) 56.52% of patients with lung cancer were diagnosed before AIS, and the peak of AIS attack was 1–6 months after the diagnosis of lung cancer. (3) The independent risk factors of LCA-AIS were CYFRA-211 (OR 1.070; 95% confidence interval 1.005, 1.139; *p* = 0.035), TT (OR 1.275; 95% confidence interval 1.089, 1.493; *p* = 0.003), and Hct (OR 0.878; 95% confidence interval 0.779, 0.990; *p* = 0.034), making ROC curve, suggesting the area under the curve is 0.871. (4) The neurological deficit of patients in the LCA-AIS group was similar to the simple AIS group and could not be identified by the severity of neurological deficits. (5) The median survival time of LCA-AIS group patients was five months (95% confidence interval 3.796, 6.204). There were statistical differences in survival time between LCA-AIS group and simple AIS group patients (*p* < 0.001).

**Conclusions:**

The interaction between lung cancer and AIS may shorten patients' life expectancy and worsen their quality of life.

## 1. Introduction

In 1865, Armand Trousseau first reported a causal relationship between occult gastric cancer and thrombosis and then was named Trousseau syndrome. With the deepening clinical and basic researches, the concept of Trousseau syndrome is widely used for cancer-related thrombosis. Acute ischemic stroke (AIS) is one of the most important diseases related to thrombosis in the brain. Some studies in various countries have found that the incidence of AIS is significantly higher in cancer patients than in noncancer patients [1–7], but the specific causes are not clear. Most of the authors suggested in published articles that the biological indicators we should focus on cancer-associated AIS patients were D-dimers and carbohydrate tumor markers [8–12]. However, the research concerning clinical characteristics, risk factors, pathogenesis, and survival situation of cancer-associated AIS patients were rare. According to the National Cancer Center report, the highest incidence and mortality of cancer in China was of lung cancer [13]. In order to further understand the AIS characteristics and risk factors in lung cancer-associated acute ischemic stroke (LCA-AIS) patients, we collected the clinical data of those patients and explored its clinical features, risk factors, and prognosis.

## 2. Information and Methods

### 2.1. Clinical Information

#### 2.1.1. Diagnostic Criteria

(1) Lung cancer diagnostic criteria: the diagnosis of lung cancer based on pathological outcome. (2) AIS diagnostic criteria: the diagnosis of AIS was based on the American Heart Association diagnostic criteria for cerebral infarction and combined with the results of brain MRI examination. (3) LCA-AIS diagnostic criteria: (1) comply with the diagnostic criteria for lung cancer and AIS; (2) the onset time of AIS is later than lung cancer; (3) no common risk disease of AIS; and (4) through clinical discussion by two neurology attending physicians, AIS pathogenesis was considered to be related to lung cancer.

#### 2.1.2. Inclusion Criteria and Methods

A total of three groups of patients were divided into an LCA-AIS group, simple lung cancer group, and simple AIS group. 1. LCA-AIS group inclusion criteria: (1) patients with previous history of lung cancer and then attacked AIS or patients with AIS attack were found to have lung cancer during hospitalization; (2) no common risk diseases of AIS; (3) TOAST classification was classified “SUE;” and (4) discussed by two clinical attending physicians and considered that the cause of AIS was associated with lung cancer. (2) Simple lung cancer group inclusion criteria: (1) patients with lung cancer admitted to the hospital at the same time and had similar age as the LCA-AIS group patients; (2) no ischemic brain infarction according to the brain MRI result; and (3) After 6 months, the brain MRI was checked again, and no ischemic brain infarction lesions were found either. (3) Simple AIS group inclusion criteria: (1) patients with AIS admitted to the hospital at the same time and with similar age as LCA-AIS group patients; (2) after completing the relevant examination after hospitalization, it is clear that the patient did not have any cancer. Clinical primary data and laboratory results were compared with hospitalization date- (including three days before and after) and age- (difference of less than one year) matched patients in the LCA-AIS group, simple lung cancer group, and simple AIS group.

#### 2.1.3. Data Collection

Patients' necessary information (including gender, age, underlying diseases, and tumor stage), laboratory results (including blood routine, blood coagulation, biochemistry, tumor markers, and tumor histopathology reports), imaging reports (including neck vascular ultrasound, brain MRI, and brain MRA), neurological deficits (mRS score), and follow-up information were collected.

### 2.2. Statistical Methods

All data were statistically analyzed using SPSS 20.0 software, and the level of significance was tested as *p* < 0.05. Continuous variables with normal distribution were reported using mean and standard deviation, and the difference between the two groups was compared using paired *t*-test. Continuous variables with abnormal distribution were reported using median and interquartile range, and the difference between the two paired groups was compared using the Wilcoxon test. Categorical variables were reported with number and percentage, and the difference between the two groups was compared with the chi-square test. Independent risk factors analysis used logistic regression. Firstly, one-factor regression analysis of independent variables was performed, the entry method was used to calculate the *p* value, and the factors that have *p* value less than 0.1 were collected into the final regression equation. The application of the entry method was used to calculate the final *p* value, OR value, and 95% confidence interval. Survival curves generated by the Kaplan–Meier method and logarithmic rank method were used to compare the difference between the survival curves in two groups.

## 3. Result

### 3.1. Basic Information

We enrolled 46 patients in each LCA-AIS group, simple lung cancer group, and simple AIS group during January 2014 to December 2018 in Hainan Hospital of Chinese PLA General Hospital. There were 29 males and 17 females in the LCA-AIS group, with an average age of 65.80 ± 11.75 years. There were 25 males and 21 females in simple lung cancer group, with an average age of 65.59 ± 11.73 years. There were 28 males and 18 females in the simple AIS group, with an average age of 65.80 ± 11.75 years. In the LCA-AIS group, the pathology of lung cancer was more common in adenocarcinoma (69.56%), the proportion of poorly differentiated patients (78.26%) was significantly more than that in moderately differentiated or highly differentiated, and the stages of lung cancer were significantly more common in stage IV (76.08%). There was no significant difference in basic information of patients between the LCA-AIS group and simple lung cancer group Table 1.

### 3.2. Relationship between Lung Cancer and AIS Onset Time in Patients with LCA-AIS

**Figure 1:**
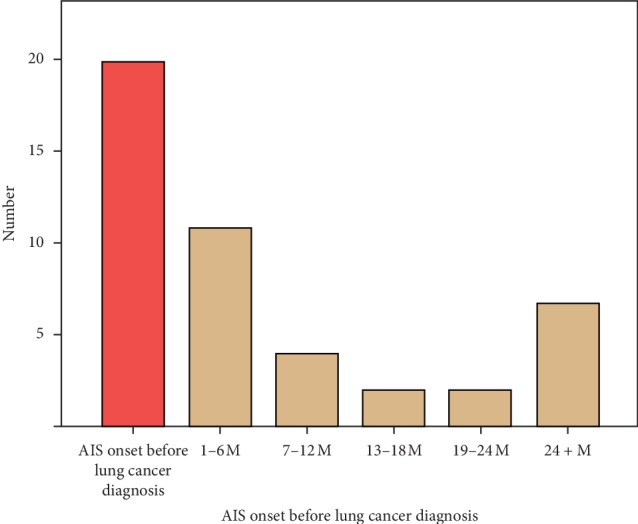
Relationship between lung cancer and AIS onset time in patients with LCA-AIS.

In the LCA-AIS group, 20 patients (43.48%) were found to attack AIS as the first onset form, and 26 patients (56.52%) attacked AIS after diagnosis of lung cancer. The peak period of AIS in patients with lung cancer appears in 1–6 months after the diagnosis of lung cancer, and then the risk of AIS attack decreased. Another peak occurred in the interval of above 24 months, combined with medical history, and it was found that those patients had lung cancer progression before the onset of AIS attack Figure 1.

### 3.3. Analysis of Laboratory Results

**Figure 2:**
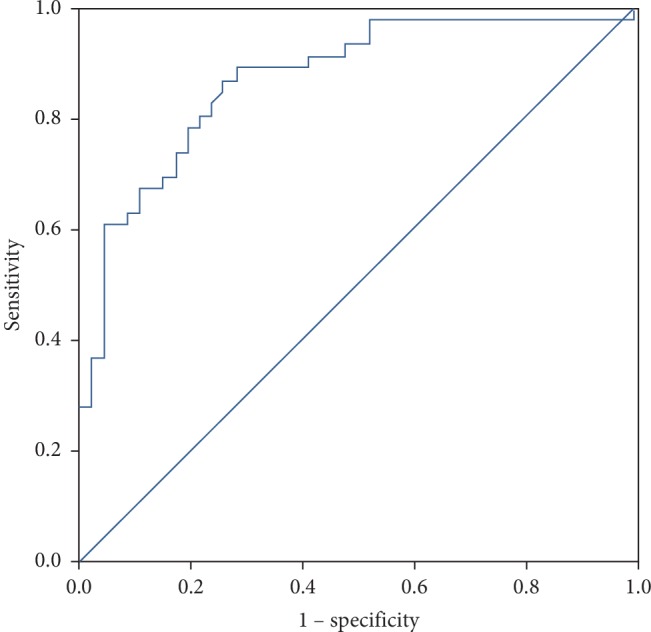
ROC curve analysis for the CYFRA-211, TT, and Hct value in the LCA-AIS group and simple lung cancer group.

The results of the patient's blood test routine, coagulation, and tumor markers were collected and analyzed by regression analysis (Table 2). The independent risk factors of LCA-AIS were CYFRA-211 (OR 1.070; 95% confidence interval 1.005, 1.139; *p*=0.035); TT (OR 1.275; 95% confidence interval 1.089, 1.493; *p*=0.003); and Hct (OR 0.878; 95% confidence interval 0.779, 0.990; *p*=0.034). There was no significant statistical difference in residual CA125 (*p*=0.243), CA153 (*p*=0.085), NSE (*p*=0.418), D-dimer (*p*=0.879), and INR (*p*=0.295). We combined three important variables (CYFRA-211; TT; Hct) into the logistic regression together to calculate the probability and made the ROC curve based on the probability we obtained. It was suggested that the area under the curve is 0.871 Figure 2.

### 3.4. Assessment of Neurological Deficits

**Figure 3:**
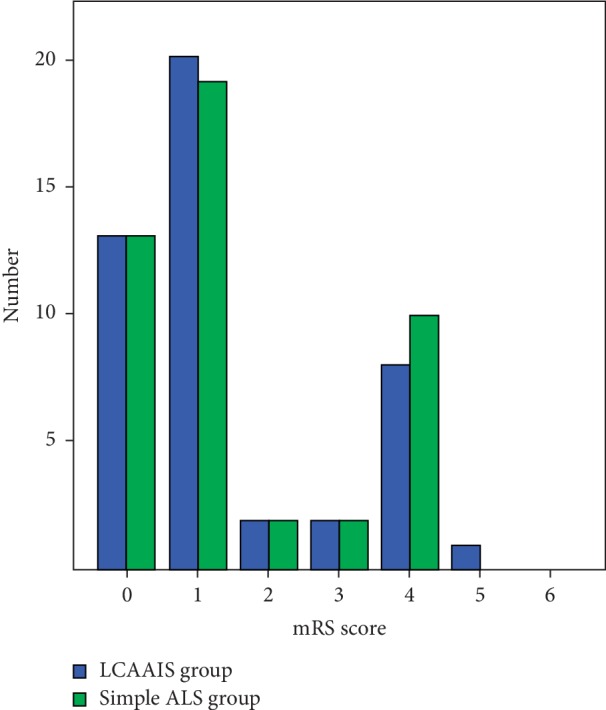
Comparison of mRS scores between the LCA-AIS group and simple AIS group.

The neurological deficits in the LCA-AIS group and the simple AIS group were analyzed by the modified Rankin Scale (mRS) score. In the LCA-AIS group, we found totally 33 patients (71.74%) with clinical neurological deficits (mRS score ≥1 point), 13 patients (28.26%) with clinical disability (mRS score ≥2 point), and 11 patients (23.91%) with moderate or above disability who need other people to help with their lives (mRS score ≥3 point). In the simple AIS group, we found also totally 33 patients (71.74%) with clinical neurological deficits (mRS score ≥1 point), 15 patients (32.61%) with clinical disability (mRS score ≥2 point), and 12 patients (26.09%) with moderate or severe disability who need other people to help with their lives (mRS score ≥3 point). The clinical neurological deficits in patients with LCA-AIS and simple AIS were mainly characterized by central facial lingual dysfunction, dysarthria, aphasia, limb dysfunction, and dyskinesia. It was found that the neurological deficits were similar, and there was no significant difference between the two groups (*p*=0.893) Figure 3.

### 3.5. Survival of Patients in the LCA-AIS Group and Simple AIS Group

**Figure 4:**
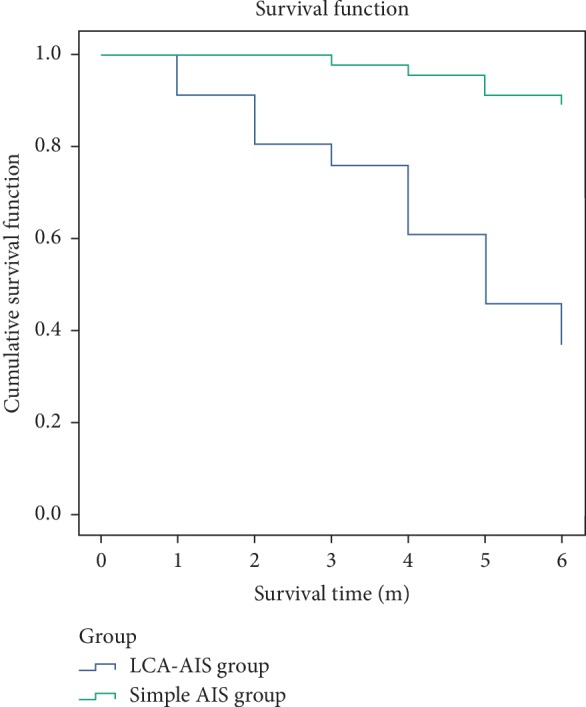
Patient survival function in the LCA-AIS group and simple AIS group.

The date of AIS attack in the LCA-AIS group was used as a starting point and death due to tumor and AIS as an endpoint. In the LCA-AIS group, it was found that 17 patients (36.95%) survived after six months, and the median survival time was five months (95% confidence interval 3.796, 6.204). In the simple AIS group, we found that 41 patients (89.13%) survived after six months. After comparing the survival function of patients in the LCA-AIS group and simple AIS group, there were statistical differences in survival time between the two groups (*p* < 0.001) Figure 4.

## 4. Discussion

Cancer and AIS have become the most common causes of death among the elderly in the world. The World Health Organization estimated that there were 18.1 million new cancer cases and 9.6 million cancer deaths worldwide in 2018 and that the number of people who have cancer worldwide is rapidly growing [14]. The relationship between cancer and AIS is now being recognized, so with the rapid increase in the number of cancer patients, we expected that the number of cancer-associated AIS would also increase year by year. A recent research indicated there was a positive association between stroke and any cancer in men and women, and the association was significant for cancers of respiratory and intrathoracic organs [15]. Several cohort studies also have found that lung cancer, colon cancer, rectal cancer, pancreatic cancer, central nervous system cancer, stomach cancer, and ovarian cancer can increase the risk of AIS attacks, but breast cancer has no significant association with increased risk of AIS [12, 16–18], suggesting the probability of causing AIS attacks is different in different types of cancer, and it is speculated that the mechanism and the indicators of AIS in different types of cancer also may be different. Because lung cancer has the highest incidence and mortality in China, this study mainly aimed at LCA-AIS patients.

Our study found that AIS can occur at any stage of lung cancer, and as the tumor progresses, the number of LCA-AIS patients shows an upward trend. So, we found the number of LCA-AIS patients in stage IV significantly more than in other stages. The number of LCA-AIS patients with lung adenocarcinoma cancer was found to be more than that of patients with squamous cell cancer, and pathologically poorly differentiated was significantly higher than that of moderately or highly differentiated. Adenocarcinoma cells can enhance thrombin by producing mucin and directly secrete into the blood to help make the hypercoagulable state. Meanwhile, mucin also can interact with specific cell adhesion molecules on endothelial cells, platelets, and lymphocytes to induce the formation of platelet-rich microthrombin [19].

Our study found that AIS may be a unique precursor to occult lung cancer. 43.48% of patients in the LCA-AIS group were found to have lung cancer due to attack of AIS in our research. In the LCA-AIS patients group, the peak incidence of AIS was 1–6 months after the diagnosis of lung cancer; the risk would persist, but it shows a downward trend. The results suggested that for patients with newly diagnosed lung cancer, the brain MRI should be closely improved within the first six months to determine whether or not attacked AIS.

As mentioned before, the mechanism and the indicators of AIS in different types of cancer may be different. However, the research focused on the risk factors of LCA-AIS patients is sporadic at present. Xie et al. [8] found that LCA-AIS patients had higher blood levels of CA125 and CA199 compared with simple lung cancer patients, but the study involved few related tumor markers and cannot completely exclude the possibility of simple lung cancer group patients occur AIS in short period after recruitment. There are few reports suggesting that independent risk factors of multiple types of cancer-associated AIS patients are D-dimer and carbohydrate tumor markers [1, 20–22]. Considering the reason, we thought it may be related to the difference in the mechanism, pathological type, and different cancers. The statistical analysis of multiple types of tumors may result in an offset, which may result in different results.

CYFRA-211 is a soluble fragment of cytokeratin 19, which has positive significance for the diagnosis of non-small-cell lung cancer. The degree of elevated concentration is clinically suggestive of the progression and severity of lung cancer and can also predict the prognosis of lung cancer patients. Our study found that CYFRA-211 was an independent risk factor for LCA-AIS patients. For every 1 ng/ml increase in concentration, the risk of AIS attacks increases by 7%. Cancer cells can activate the body's coagulation mechanism by releasing many tissue factors, cancer-associated procoagulant, and cyokines, and cancer cells also can interact with platelets to release ADP, arachidonic acid, 5-HT, and other substances to promote blood coagulation. Eventually, the body will be hypercoagulable [23–27]. Bang's recent research showed circulating tumor extracellular vesicles, especially in adenocarcinoma patients, also played a vital role in cancer-associated thrombosis and tumor progression [28, 29]. However, our study also found that TT was also an independent risk factor for LCA-AIS patients. We found that the risk of AIS increased by 8.9% for every 1s prolong of TT. TT prolongation indicates that there is hyperfibrinolysis in the body. Considering the LCA-AIS group, the fibrinolytic mechanism was activated, and the body was in the secondary fibrinolysis stage. Therefore, it is speculated that the pathogenesis of LCA-AIS may not only be related to the activation of the blood coagulation mechanism but also related to the activation of the fibrinolysis mechanism, which ultimately leads to the onset of AIS. Hct refers to the ratio of red blood cells to total blood volume and is an indicator that reflects the number, size, and volume of red blood cells. It can directly affect the ability of red blood cells to carry oxygen transport and also plays an essential role in regulating blood flow. The role of Hct in noncancer patients attack acute cardiovascular and cerebrovascular events remains controversial. However, in our study, we found a negative correlation between Hct and LCA-AIS, with every 1% increase in Hct and 12.2% reduction in AIS risk. Hypoglobinemia due to cancer progression led to impaired oxygen transport, while the brain was highly dependent on oxygen supply and had no oxygen storage capacity. Therefore, when the red blood cells were insufficiently supplied with oxygen, the AIS may attack.

In terms of neurological deficits, there was no significant difference between the patients in the LCA-AIS group and those in the simple AIS group, although almost 30% of patients have moderate disability in each group. The patients with localized neurological deficits were mainly characterized, and the symptoms and degrees of neurological deficits were very similar between LCA-AIS patients and simple AIS patients. Patients with LCA-AIS are faced with multiple risks. The progression of lung cancer and the complications of AIS had a dual impact on patient survival time. Our study found that the median survival time of LCA-AIS patients was only five months. Meanwhile, nearly 90% of patients in the simple AIS group survived later than six months.

## 5. Conclusion

The interaction between lung cancer and AIS may shorten patients' life expectancy and worsen their quality of life.

## Figures and Tables

**Table 1:** Basic information of patients in the LCA-AIS group and simple lung cancer group.

	LCA-AIS group	Simple lung cancer group	*p* value
Male	29 (63.04%)	25 (54.38%)	0.397
Average age	65.80 ± 11.75	65.59 ± 11.73	0.133
Smoke	7 (15.22%)	11 (23.91%)	0.293
Hypertension	0 (0%)	3 (6.52%)	0.242
Diabetes	0 (0%)	2 (4.35%)	0.495
Coronary heart disease	0 (0%)	3 (6.52%)	0.242
Atrial fibrillation	0 (0%)	1 (2.17%)	1.000
Lung cancer pathology			
Adenocarcinoma	32 (69.56%)	34 (73.91%)	0.886
Squamous cell carcinoma	12 (26.08%)	10 (21.73%)	
Small-cell lung cancer	2 (4.34%)	2 (4.34%)	
Adenocarcinoma			
Poorly differentiated	24 (52.17%)	25 (54.34%)	0.861
Moderately differentiated	7 (15.21%)	7 (15.21%)	
Highly differentiated	1 (2.17%)	2 (4.34%)	
Squamous cell carcinoma			
Poorly differentiated	12 (26.08%)	9 (19.56%)	0.455
Moderately differentiated	0 (0%)	1 (2.17%)	
Highly differentiated	0 (0%)	0 (0%)	
Lung cancer staging			
I	3 (6.52%)	4 (8.69%)	0.978
II	3 (6.52%)	3 (6.52%)	
III	3 (6.52%)	5 (10.86%)	
IV	35 (76.08%)	32 (69.56%)	
Limit period			
(small-cell lung cancer)	1 (2.17%)	1 (2.17%)	
Extensive period			
(small-cell lung cancer)	1 (2.17%)	1 (2.17%)	

**Table 2:** One-factor regression analysis of various laboratory indicators between the LCA-AIS group and simple lung cancer group.

		LCA-AIS group	Simple lung cancer group	*p* value	OR	95% CI
CEA		12.81 (3.79, 81.30)	5.24 (2.14, 44.50)	0.955	1.000	0.999, 1.001
AFP		2.34 (1.88, 3.32)	2.91 (2.45, 4.13)	0.893	1.011	0.865, 1.181
CA125		130.95 (31.00, 365.38)	25.52 (11.37, 72.59)	0.031	1.002	1.000, 1.004
CA199		27.72 (11.32, 80.45)	18.37 (8.19, 37.52)	0.123	1.002	0.999, 1.005
CA153		31.44 (18.13, 92.82)	17.76 (11.75, 23.82)	0.06	1.006	1.000, 1.013
CA724		4.72 (2.17, 17.66)	2.18 (1.06, 4.99)	0.141	1.011	0.996, 1.025
CYFRA-211		14.90 (5.78, 41.37)	3.05 (1.94, 5.29)	<0.001	1.099	1.044, 1.156
NSE		18.18 (13.43, 25.88)	13.52 (11.16, 14.99)	0.03	1.051	1.055, 1.100
D-dimer		1736.50 (434.00, 4240.5)	368.50 (100.50, 1096.75)	0.001	1.001	1.000, 1.001
INR		1.10 ± 0.11	1.03 ± 0.09	0.005	552.169	6.835, 44610.332
PT		13.37 ± 1.63	12.56 ± 1.63	0.023	1.364	1.044, 1.782
PTA		89.67 ± 15.55	97.82 ± 15.39	0.017	0.966	0.939, 0.994
TT		19.72 ± 3.97	16.70 ± 4.08	0.001	1.199	1.075, 1.337
FIB		3.59 ± 1.81	3.74 ± 1.14	0.631	0.935	0.712, 1.229
APTT		32.41 ± 7.30	31.12 ± 5.32	0.337	1.033	0.967, 1.104
Hb		110.73 ± 22.67	123.89 ± 17.31	0.004	0.968	0.946, 0.990
RBC		3.99 ± 0.82	4.36 ± 0.48	0.015	0.431	0.218, 0.852
Hct		33 ± 6	37 ± 4	0.002	0.878	0.808, 0.954
MCV		85.44 ± 9.12	87.68 ± 6.73	0.188	0.964	0.913, 1.018
MCH		27.87 ± 3.53	28.58 ± 2.84	0.297	0.932	0.816, 1.064
MCHC		326.5 ± 14.72	325.50 ± 14.14	0.737	1.005	0.977, 1.034
PLT		226.15 ± 99.91	255.00 ± 87.87	0.149	0.997	0.992, 1.001

## Data Availability

The data used to support the findings of this study are available from the corresponding author upon reasonable request.

## References

[1] Bang O. Y., Seok J. M., Kim S. G. (2011). Ischemic stroke and cancer: stroke severely impacts cancer patients, while cancer increases the number of strokes. *Journal of Clinical Neurology*.

[2] Chen P.-C., Muo C.-H., Lee Y.-T., Yu Y.-H., Sung F.-C. (2011). Lung cancer and incidence of stroke. *Stroke*.

[3] Zöller B., Ji J., Sundquist J., Sundquist K. (2012). Risk of haemorrhagic and ischaemic stroke in patients with cancer: a nationwide follow-up study from Sweden. *European Journal of Cancer*.

[4] Navi B. B., Reiner A. S., Kamel H. (2015). Association between incident cancer and subsequent stroke. *Annals of Neurology*.

[5] Navi B. B., Reiner A. S., Kamel H. (2017). Risk of arterial thromboembolism in patients with cancer. *Journal of the American College of Cardiology*.

[6] Navi B. B., Howard G., Howard V. J. (2018). New diagnosis of cancer and the risk of subsequent cerebrovascular events. *Neurology*.

[7] Jang H. S., Choi J., Shin J. (2019). The long-term effect of cancer on incident stroke: a nationwide population-based cohort study in Korea. *Frontiers in Neurology*.

[8] Xie X., Chen L., Zeng J. (2016). Clinical features and biological markers of lung cancer-associated stroke. *Journal of International Medical Research*.

[9] Qin Q.-X., Cheng X.-M., Lu L.-Z. (2018). Biomarkers and potential pathogenesis of colorectal cancer-related ischemic stroke. *World Journal of Gastroenterology*.

[10] Wang J.-y., Zhang G.-j., Zhuo S.-x. (2018). D-dimer >2.785 *μ*g/ml and multiple infarcts ≥3 vascular territories are two characteristics of identifying cancer-associated ischemic stroke patients. *Neurological Research*.

[11] Nam K.-W., Kim C. K., An T. J. (2017). D-dimer as a predictor of early neurologic deterioration in cryptogenic stroke with active cancer. *European Journal of Neurology*.

[12] Nam K. W., Kim C. K., Kim T. J. (2017). Predictors of 30-day mortality and the risk of recurrent systemic thromboembolism in cancer patients suffering acute ischemic stroke. *PLoS One*.

[13] Zheng R. S., Sun K. X., Zhang S. W. (2019). Report of cancer epidemiology in China, 2015. *Chinese Journal of Oncology*.

[14] Bray F., Ferlay J., Soerjomataram I., Siegel R. L., Torre L. A., Jemal A. (2018). Global cancer statistics 2018: GLOBOCAN estimates of incidence and mortality worldwide for 36 cancers in 185 countries. *CA: A Cancer Journal for Clinicians*.

[15] Jacob L., Kostev K. (2019). Cancer risk in stroke survivors followed for up to 10 years in general practices in Germany. *Journal of Cancer Research and Clinical Oncology*.

[16] Grazioli S., Paciaroni M., Agnelli G. (2018). Cancer-associated ischemic stroke: a retrospective multicentre cohort study. *Thrombosis Research*.

[17] Selvik H. A., Bjerkreim A. T., Thomassen L. (2018). When to screen ischaemic stroke patients for cancer. *Cerebrovascular Diseases*.

[18] Selvik H. A., Thomassen L., Bjerkreim A. T., Næss H. (2015). Cancer-associated stroke: the bergen NORSTROKE study. *Cerebrovascular Diseases Extra*.

[19] Dearborn J. L., Urrutia V. C., Zeiler S. R. (2014). Stroke and cancer: a complicated relationship. *Journal of Neurology & Translational Neuroscience*.

[20] Lee E.-J., Nah H.-W., Kwon J.-Y., Kang D.-W., Kwon S. U., Kim J. S. (2014). Ischemic stroke in patients with cancer: is it different from usual strokes?. *International Journal of Stroke*.

[21] Aarnio K., Joensuu H., Haapaniemi E. (2015). Cancer in young adults with ischemic stroke. *Stroke*.

[22] Chen Y., Zeng J., Xie X. (2015). Clinical features of systemic cancer patients with acute cerebral infarction and its underlying pathogenesis. *International Journal of Clinical and Experimental Medicine*.

[23] Long H., Qin K., Chen J. (2018). Biomarkers of gastric cancer-related ischemic stroke and its underlying pathogenesis. *Medicine (Baltimore)*.

[24] Chan P. C., Chang W. L., Hsu M. H. (2018). Higher stroke incidence in the patients with pancreatic cancer: a nation-based cohort study in Taiwan. *Medicine*.

[25] Grisold W., Oberndorfer S., Struhal W. (2009). Stroke and cancer: a review. *Acta Neurologica Scandinavica*.

[26] Cestari D. M., Weine D. M., Panageas K. S., Segal A. Z., DeAngelis L. M. (2004). Stroke in patients with cancer: incidence and etiology. *Neurology*.

[27] Guerrero B., López M. (2015). Overview of the coagulation system and laboratory tests for its study. *Investigación Clínica*.

[28] Chung J. W., Cho Y. H., Ahn M. J. (2019). Association of cancer cell type and extracellular vesicles with coagulopathy in patients with lung cancer and stroke. *Frontiers in Neurology*.

[29] Bang O. Y., Chung J. W., Lee M. J. (2016). Cancer cell-derived extracellular vesicles are associated with coagulopathy causing ischemic stroke via tissue factor independent way: the OASIS-CANCER Study. *PLoS One*.

